# Personalized Body Constitution Inquiry Based on Machine Learning

**DOI:** 10.1155/2020/8834465

**Published:** 2020-11-12

**Authors:** Baochao Fan, Yanghui Li, Guihua Wen, Yan Ren, Yantong Lu, Ziying Wang, Yuan Zhang, Changjun Wang

**Affiliations:** ^1^Guangzhou University of Chinese Medicine, Guangzhou, China; ^2^Guangdong Provincial People's Hospital, Guangdong Academy of Medical Sciences, Guangdong Geriatric Institute, Guangzhou, China; ^3^South China University of Technology, Guangzhou, China; ^4^Southern Medical University, Guangzhou, China

## Abstract

**Background:**

Body constitution (BC) is the abstract concept indicating the state of a person's health in Traditional Chinese Medicine (TCM). The doctor identifies the body constitution of the patient through inspection and inquiry. Previous research simulates doctors to identify BC types according to a patient's objective physical indicators. However, the lack of subjective feeling information can reduce the accuracy of the machine to imitate the doctor's diagnosis. The Constitution in Chinese Medicine Questionnaire (CCMQ) is used to collect subjective information but suffers from low acquisition efficiency.

**Methods:**

This paper presents a personalized body constitution inquiry method based on a machine learning technique. It employs a random generator, a feature extractor, and a classifier to simulate the doctor inquiry and generate a personalized questionnaire. Specifically, the feature extractor evaluates and sorts the question of the constitution in the CCMQ based on the recognition results of the tongue coating image of patients. The sorted questions and relevant BC label are inputted into the classifier; the best questions are screened out for patients.

**Results:**

The experimental results show that our method can select personalized questions from the CCMQ for the patients, significantly reducing the time and the number of questions to answer. It also improves the accuracy of recognizing BC. Compared with the CCMQ, patients had 68.3% fewer questions to answer and the time occupied by answering is reduced by 80.3%.

**Conclusions:**

The proposed method can simulate the doctor's inquiry and pick out personalized questions for patients. It can act as auxiliary diagnosis tools to collect subjective patient feelings and help make further judgments on the patient's BC types.

## 1. Introduction

Based on the innate inheritance of the human body and the influence of acquired factors, Body Constitution (BC) is comprehensively expressed through various aspects such as psychological state, viscera function, metabolic function, and human morphology. BC is an inherent characteristic of a relatively stable human body [1]. BC type of a patient can help doctors understand the patients' health status and disease outcome, and then develop the targeted prevention, treatment, and rehabilitation programs. There are nine BC types according to the constitution theory in Traditional Chinese Medicine (TCM). They are Balanced Constitution, Qi-deficient Constitution, Yang-deficient Constitution, Yin-deficient Constitution, Phlegm-dampness Constitution, Damp-heat Constitution, Stagnant Blood Constitution, Stagnant Qi Constitution, and Inherited Special Constitution, where balanced constitution is well health status and the others are pathological [1]. BC identification is the research foundation of the constitution in Chinese medicine theory. Wang et al. developed the Constitution in Chinese Medicine Questionnaire (CCMQ) and the body constitution identification standard, which integrates multiple disciplines such as epidemiology, immunology, genetics, and mathematical statistics [2]. In the clinic, doctors carry on the BC Identification to the patient through the inspection and the inquiry result. The content of the inquiry comes from the questions in CCMQ [3, 4].

Artificial intelligence has integrated into the medical field development over the past decade. For TCM, the combination of emerging technologies and traditional theories has created several high-tech medical devices [5] that assist TCM doctors in judging diseases based on the intuitive data information, and even exploiting the rules of TCM from the collecting medical data [6] and simulate how the doctor diagnoses and prescribes. As for BC identification, researchers judge BC types by using machine learning to analyze the characteristic of the pulse [7], tongue [8], and face [9], which simulates the doctor's inspection. However, from the medical point of view, objective and subjective factors need to be considered comprehensively in clinical diagnosis. The image-BC identification technology analyzes the objective information of patients, while the subjective feeling information of patients is not analyzed. The lack of subjective feeling information can reduce the accuracy of the machine to imitate the doctor's diagnosis. Therefore, we propose to analyze the subjective feeling information of patients by adding questionnaire data, which simulate the doctor inquiry. Specifically, we use the CCMQ to collect the patient's subjective feelings to make further judgments on their BC types. However, CCMQ has some disadvantages during collecting data. For example, the number of questions is large; thus, patients will spend a long time on it. Many patients will be impatient when filling out the questionnaire, which will affect their choices. Patients in hospitals and community clinics often worry about their illness or experience anxiety while waiting for a doctor's consultation, and therefore they do not have the patience to answer more questions, which leads to deviations in their BC identification. In summary, it is not realistic to use the full questionnaire as the basis for collecting data. In clinical practice, the doctor narrows the range of diagnoses by inspection and asks personalized questions according to the results of inspection and determines the diagnosis results finally. Therefore, how to push personalized and precise questions for each patient is the key to simulate the doctor inquiry. This paper proposes a BC identification method based on a random generation algorithm, feature selection algorithm, and classification methods. This method uses feature selection and classification technology for screening of the CCMQ problems and pushes personalized questions to patients according to the results of the inspection. We provide a complete questionnaire and evaluation criteria in the appendix.

**Figure 1:**
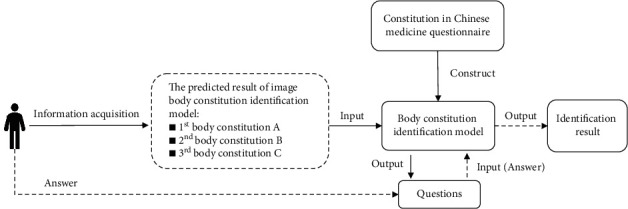
A body constitution identification method.

**Figure 2:**
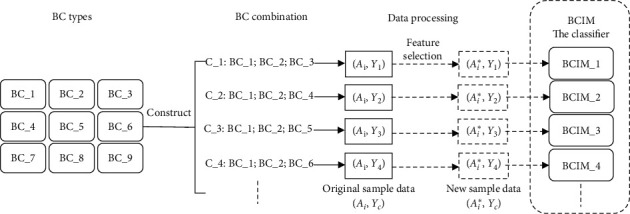
Construction of the BC combination and the BCIM. **BC_**: Nine BC types; **C_: BC_1; BC_2; BC_3**: The BC combination number and the BC types; “⟶”: The BC combination matches the corresponding data in the original sample data (*A*_*i*_, *y*_*i*_) to obtain its own original sample data (*A*_*i*_, *Y*_*c*_); “⟶”: The feature selection algorithm measures the importance score *W* of each question in each BC combination. The filtered questions collate the original sample data (*A*_*i*_, *Y*_*c*_) to obtain the new sample data (*A*_*i*_^*∗*^, *Y*_*c*_); “⟶”: Based on the new sample data (*A*_*i*_^*∗*^, *Y*_*c*_), the identification model is constructed through the classifier; **BCIM_:** The identification model corresponding to each BC combination; they are all part of the BCIM.

The main contributions of this paper are as follows:

1. Inspired by the doctor's diagnosis process, the questions of the CCMQ are introduced to quantify the patient's subjective feelings to improve the accuracy of recognizing BC automatically. The CCMQ has the disadvantages of low acquisition efficiency and susceptibility to interference. We use feature selection algorithms to achieve the selection of personalized questions, which can improve collection efficiency.
2. In order to simulate the doctor inquiry, we propose a BC identification method based on a random generation algorithm, feature selection algorithm, and classification methods, and construct a body constitution identification model (BCIM) (Figure 1).
3. We adjusted the output range of the image-BC (identifying body constitution by the human body image) identification results to the first three, which provides the identification scope to make a further judgment (Figure 1). In order for the BCIM to be able to handle all output situations of the image-BC recognition method, we combine nine BC types into the combinations each of which contains three different BC without considering the combination order. Each BC combination represents an output situation. According to the BC types contained in each combination, original sample data are processed and corresponding BCIM are constructed (Figure 2).
4. Using the doctor's judgments as references for comparison with the image-BC identification method, the accuracy of our method is improved by 25.8%. Compared with the CCMQ, patients had 68.3% fewer questions to answer than it, and the time occupied by answering is reduced by 80.3%.

## 2. Related Work

Feature selection [10] is a key technology in processing high-dimensional data in computer pattern recognition tasks. Its function is to obtain as small a subset of features as possible to improve the effect of classification, clustering, and retrieval without significantly reducing the classification effect. Therefore, feature selection algorithms are often used in conjunction with classifiers. There is a huge amount of data in the medical field, and this will continue to grow as new technologies become available. Faced with an increasing amount of information and data types, the feature selection algorithm and classifier play a vital role in helping doctors obtain disease information most relevant to disease diagnosis from large amounts of data. In the past ten years, the application of feature selection and classifiers has been deeply applied in various disciplines and in the medical field [11]. For example, biomicroarray data analysis [12], biomedical signal processing [13–15], medical imaging [16, 17], medical modeling [18], disease diagnosis classification [19], and medical diagnostic system development [20] have achieved innovative breakthroughs. Such breakthroughs have been especially seen in big data biological information processing [21] and big data information mining [22]. A doctor can predict a human's birth [23] and death [24] from physiological indicators through screening by feature selection technology. For drug development, feature selection and classifiers are used to predict functional classes of newly generated protein sequences [25] and protein inhibitors and substrates [26]. In clinical tests, they are used to predict the rate of amyloid aggregation [27] and the production of high antivasoactive peptides [28]. In summary, the main idea of these studies is to narrow the feature set by filtering the relevant data through a combination of single or multiple feature algorithms [29] and then enter the new feature set into the classifier to classify and find the indicator that is most closely related to the disease. At present, there are few studies on the application of machine learning methods in medical questionnaires. Most of them are concentrated on using a genetic algorithm (GA) to simplify questionnaires [30–32]. In this paper, we use the feature selection method and classifier technology to mine the characteristics of the scale questions and screen out highly targeted questions. The patient only needs to answer targeted questions about them to complete the data collection.

## 3. Materials and Methods

### 3.1. Task Description

The task of this paper is to build a BCIM based on the questionnaire options and judgment results of CCMQ. In our method, the BCIM takes the result of image-BC identification as input and outputs the corresponding CCMQ questions for the patient to answer. Finally, the BCIM judges the BC type based on the patient's answer (Figure 1).

### 3.2. Data Collection

The dataset for this study was generated by the random generation algorithm. We simulated the patient's answer by randomly selecting the options, and each simulation result was taken as a sample of the dataset. In order to facilitate the collection of data in the dataset, we combined two questions (60_1 and 60_2 in the CCMQ) that need to be answered according to gender attributes. We randomly assign a score of 1–5 to the 60 questions *Q* = {*q*_1_, *q*_2_,…, *q*_60_} to generate *m* different samples *A*_*i*_ = {*A*_1_, *A*_2_,…, *A*_*m*_}. The ith sample in *A* is denoted as *A*_*i*_. *A*_*i*_ = {*a*_1_, *a*_2_,…, *a*_60_}, *a*_*j*_, 1 ≤ *j* ≤ 60, and *a*_*j*_ ∈ [1,2,3,4,5] denotes the patient's score when answering the jth question. According to the CCMQ's judgment method and standard, we calculate and distinguish the BC types of each answer. There are *m* judging results *y*_*i*_ = {*y*_1_, *y*_2_,…, *y*_*m*_}. Given the answers of the *i*th sample, we apply the questions-based body constitution algorithm proposed in the CCMQ to get the BC types *y*_*i*_ of the *i*th sample. Finally, we construct the original dataset of *m* samples (*A*_*i*_, *y*_*i*_) = {(*A*_1_, *y*_1_), (*A*_2_, *y*_2_),…, (*A*_*m*_, *y*_*m*_)}.

### 3.3. Construction of the BC Combination

In order to add questionnaire data to BC identification, we propose to construct the BC combination. In clinical practice, doctors narrow the range of diagnoses by inspection, but the final diagnosis still needs the assistance of inquiry. The output process of the image-BC identification method [7–9] is to output the probabilities of all types of the constitution first, and then selects the category with the highest probability as the judgment result. Therefore, in this study, we adjusted the output range of the image-BC identification results in the first three, which provides the identification scope for BCIM to make a further judgment (Figure 1). During the BCIM construction, in order to consider all the possible output situations of the image-BC recognition method, we combine nine BC types into the combinations, each of which contains three different BC without considering the combination order. According to the BC type contained in each combination, the original sample data is merged to construct the corresponding BCIM (Figure 2). It means that each BC combination has its own original sample data (*A*_*i*_, *Y*_*c*_). *y*_*i*_ ∈ *Y*_*c*_ (*Y*_*c*_ represents the cth BC combination).

### 3.4. Screening the Representative Problems for BC Identification

Take the BC combination as a unit; the feature selection algorithm screens representative problems to build a dataset for constructing the BCIM. The feature selection algorithm (FS) measures the importance score *W* of each question in the BC combination, which can be abstracted as the following function:

(1) $$\begin{array}{c} \text{FS}\left(A_{i},Y_{c}\right)=W=\left\{W_{1},W_{2},\ldots ,W_{60}\right\}. \end{array}$$

We set the number *k* of filtered questions 1 ≤ *k* ≤ 60. We pick the top *k* questions with the highest importance scores from the original questions *Q* = {*q*_1_, *q*_2_,…, *q*_60_} contained in each the BC combination. Next, we combine them into a new problem set *Q*_*k*_^*∗*^ = {*q*_1_^*∗*^, *q*_2_^*∗*^},…, *q*_*k*_^*∗*^. This means that each BC combination has a specific new problem set *Q*_*k*_^*∗*^. The new question set is the set of questions that the patient needs to answer.

### 3.5. Construction of the BCIM

The new problem set *Q*^*∗*^ obtained by the feature selection algorithm is a subset of original questions *Q*, while the assessment method in the CCMQ needs the answer of all original questions. Therefore, the assessment method in the CCMQ is not applicable to the new problem set *Q*^*∗*^. In order to identify the corresponding BC types from it, we construct a BCIM to identify BC based on *Q*^*∗*^.

#### 3.5.1. Processing Training Data

The original sample data (*A*_*i*_, *Y*_*c*_) is filtered by the new question set *Q*^*∗*^ to get the new sample data (*A*_*i*_^*∗*^, *Y*_*c*_). The algorithm is summarized as below Algorithm 1.

#### 3.5.2. Construction and Training of the BCIM

For training BCIM, the answers *A*_*i*_^*∗*^ of the new question set *Q*^*∗*^ in the new sample data (*A*_*i*_^*∗*^, *Y*_*c*_), 1 < = *i* < = *m* are input into the classifier as the features of the classifier, and the BC *y*_*i*_(*y*_*i*_ ∈ *Y*_*c*_) is the output of the classifier. The model is continuously updated through iterative training. The operation can be abstracted as the following function:

(2) $$\begin{array}{c} y_{i}=\text{classifier}\left(A_{i}^{*}\right). \end{array}$$

## 4. Results

### 4.1. Dataset

The dataset of the BCIM is collected from the randomly generated samples. The dataset can be organized into the following two parts:

1. *Original Dataset*(*A*_*i*_, *y*_*i*_). For these 60 questions in the CCMQ, the randomly generated algorithm is used to generate 1,000,000 different answers, and each answer is marked with the BC type according to the CCMQ's judgment method. A detailed description of the data is shown in Table 1.
2. *The Dataset*(*A*_*i*_*∗Y*_*c*_)*for Training the BCIM*. The BCIM is constructed based on the BC combination. Nine BC types are combined into combinations containing three different BC types, resulting in a total of 84 = *C*_9_^3^ BC combinations. The feature selection algorithm is used to screen out the top *k*, questions with the highest importance score in each BC combination. These questions are combined with options and BC labels in the original dataset to obtain a dataset for training the BCIM. There are nine constitution labels in the dataset, including Balanced Constitution, Qi-deficient Constitution, Yang-deficient Constitution, Yin-deficient Constitution, Phlegm-dampness Constitution, Damp-heat Constitution, Stagnant Blood Constitution, Stagnant Qi Constitution, and Inherited Special Constitution.

### 4.2. Experimental Setup

Filter is a feature selection algorithm that focuses on the general characteristics of the data and independent of the classifier. According to the filter's ability to score the features of each dimension, we use the chi-squared stats algorithm to evaluate the importance score of each combination of questions and screen them. Models based on linear discriminate analysis (LDA), the artificial neural network (ANN), k-nearest neighbor (k-NN), random forest (RF), and support vector machine (SVM) are constructed and run for identifying BC.

### 4.3. Evaluation Metrics

In order to evaluate the identification performances of the five aforementioned models, we use a 5-fold cross-validation method to train and evaluate the models. The new sample data (*A*_*i*_^*∗*^, *Y*_*c*_) of each combination are divided into five equal parts, taking 4/5 of the samples for the training set and the remaining for the test set. Each combination performs training five times and the test samples taken for each time do not overlap. Finally, the mean accuracy of five evaluation results is used as the accuracy of the model. In addition, we use the Macro-averaging (Macro-Precision, Macro-Recall, Macro-F Score) and Micro-averaging (Micro-Precision, Micro-Recall, Micro-F Score) as indicators to evaluate the multiclassification of the classifier. The classifier's response time is also an evaluation indicator, denoting the calculation speed of the model.

### 4.4. Results and Analysis

**Figure 3:**
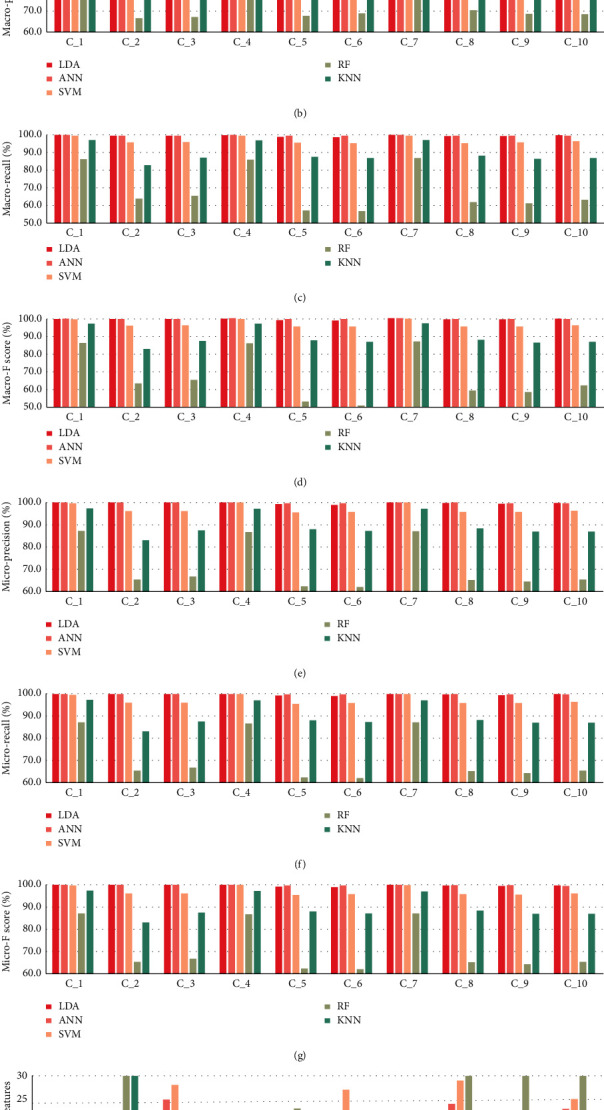
Experimental performances of different models. **A-G**: The indicator to evaluate the multiclassification of the classifier. **H**: The number of features used to construct the model when the identification accuracy of the model is highest. **C_1**: BC combination: Balanced Constitution, Yang-deficient Constitution, Qi-deficient Constitution; **C_2**: BC combination: Damp-heat Constitution, Stagnant Blood Constitution, Stagnant Qi Constitution; **C_3**: BC combination: Yang-deficient Constitution, Phlegm-dampness Constitution, Stagnant Blood Constitution; **C_4**: BC combination: Balanced Constitution, Yin-deficient Constitution, Stagnant Blood Constitution; **C_5**: BC combination: Qi-deficient Constitution, Damp-heat Constitution, Stagnant Blood Constitution; **C_6**: BC combination: Qi-deficient Constitution, Stagnant Qi Constitution, Inherited Special Constitution; **C_7**: BC combination: Balanced Constitution, Yang-deficient Constitution, Phlegm-dampness Constitution; **C_8**: BC combination: Yang-deficient Constitution, Damp-heat Constitution, Stagnant Qi Constitution; **C_9**: BC combination: Yin-deficient Constitution, Stagnant Qi Constitution, Inherited Special Constitution; **C_10**: BC combination: Phlegm-dampness Constitution, Stagnant Blood Constitution, Inherited Special Constitution.

The new sample data (*A*_*i*_^*∗*^, *Y*_*c*_) are used as training objects to compare the identification performances of the five models. We randomly select 10 BC combinations for comparison. To visually compare the performance of the classifier, we take the highest accuracy of the 5-fold as the accuracy of the model and extract the evaluation metrics data of this point. The performance is shown in Figure 3. The values obtained by LDA, ANN, and SVM models are all above 90%. This shows that identifying BC by feature selection and the classifier is feasible and effective. Notably, in the case of different BC combinations, we found that the number of features used to construct the BCIM with LDA is less than that used by other classifiers. The number of features represents the number of screened questions *k*.

**Figure 4:**
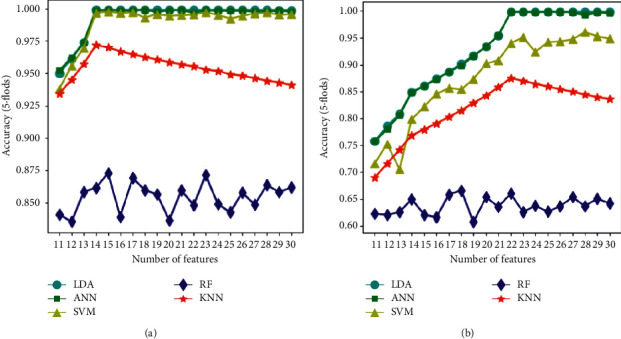
The accuracy performances of different models. (a) BC combination: Balanced Constitution, Yang-deficient Constitution, Qi-deficient Constitution. (b) BC combination: Yang-deficient Constitution, Phlegm-dampness Constitution, Stagnant Blood Constitution.

In order to further show and analyze the performance and difference of the classifier, we select two BC combinations that consist of the most common BC types in the survey [33] as examples from the comparison result. The specific data are shown in Figure 4 and Table 2. The abscissas in Figure 4 represent the number of features used to build the fitness discrimination model. The ordinate indicates the accuracy of the BCIM.

As we can see from Figure 4, the accuracy of all models would change with the number of features *k*. Models built by LDA and ANN work best, and their accuracies tend to stabilize after reaching the peak. The model built by SVM is not as good as the former two and is affected by the BC type in the combination. The accuracy of the model built by k-NN shows a downward trend after reaching the peak. This is because the increase in the number of features *k* results in an increase in the number of objects contained in the model and a decrease in the frequency of the correct category of objects. The accuracy of the model built by RF is low and shows disorderly fluctuations. This might be caused by the small number of features extracted by RF, resulting in a small subspace and low variety. In conclusion, the classification effect of LDA, ANN, and SVM models reach the experimental expectation.

In order to further compare the performance of the classifiers, we extracted the points with the highest accuracy of each model (Table 2). The value *k* represents the number of features used to construct the model making the identification accuracy of the model highest. For the Macro-averaging and the Micro-averaging, in the BC combination of Balanced Constitution, Yang-deficient Constitution, and Qi-deficient Constitution, the model built by ANN gets the highest score. All its accuracy, Micro-Precision, Micro-Recall, Micro-F Score, Macro-Recall, and Macro-F Score are 99.90%, and its Macro-Precision is 99.89%. Models built by LDA and SVM rank second and third. In the BC combination of the Yang-deficient Constitution, Phlegm-dampness Constitution, and the Stagnant Blood Constitution, the model built by LDA has the highest score. All its accuracy, Micro-Precision, Micro-Recall, Micro-F Score, Macro-Recall, and Macro-F Score are 99.84%, and the Macro-Precision is 99.83%. Models built by ANN and SVM rank second and third, respectively. Compared with the performance of the model about response time, the results of the other four classifiers except KNN are acceptable. LDA is the fastest one, whose response time is 0.009 seconds and 0.006 seconds, which means that the projection direction chosen by LDA can well classify the training data, which enables quick judgment during the test. The model built by KNN takes the most time, reaching 47 minutes, which means that the calculation efficiency of KNN is low. We believe that this is determined by the operation characteristics of KNN, which needs to calculate the distance between samples to be tested and each feature, so the calculation time of the classifier increases with the number of features.

Taken together, the performances of models built by LDA and ANN are the best and fit the purpose of our study. Although there is a slight difference between these classifiers, we can see that our proposed method can be applied to most classifiers and has high generalization. In this study, LDA is the best method to construct the BCIM if the number of questions to be answered by the patient is taken into account (Figure 3(h)).

### 4.5. Performance Comparison

In order to verify the performance of the BCIM constructed by the chi-squared stats algorithm and LDA in clinical practice application, we select the image body constitution identification model (image-BCIM) [8] as a baseline and use the doctor's judgment and judgment results of the CCMQ as reference standards. This comparison experiment compares the effect of adding inquiry on the accuracy of BC identification based on inspection. Besides, we also record the number of questions and the time of answering them, which will be compared with the CCMQ.

74 volunteers participated in this comparison experiment. During the experiment, according to image-BCIM (simulating the doctor inspection), image-BCIM + BCIM (simulating the doctor inspection and inquiry) and doctor's judgment, three methods were run for identifying the BC type of the volunteers. Another 70 volunteers performed physical examinations only by filling out the CCMQ. One example is presented in Table 3 to show the actual comparison results. The table is divided into three parts. The first part is the identification result of the image-BCIM. The second part is data from the image-BCIM + BCIM, in which we adjusted the output range of the image-BCIM identification results to the first three, in addition, the BCIM outputs questionnaire questions, identification result, and the time spent on filling and identifying. The third part is the doctor's judgment results. It should be noted that there will be multiple results in the doctor judgment, all of which are the real result [34]. The human body is often in a subhealthy state with multiple unbalanced constitutions at the same time. This is common in elderly or frail people [35]. Therefore, when the result of the image-BCIM or the image-BCIM + BCIM matches one of the judgments of the doctor, we take it as a correct one.

The comparison results with volunteers' participation are shown in Tables 4 and 5. In order to enhance the accuracy and persuasiveness of results, the evaluation results are averaged by test results. The values after “±” indicate the standard deviation of test results.

Taking the identification result of the doctor as the reference (Table 4), the accuracy rate of the image-BCIM + BCIM is 77.4%, which is 25.8% higher than that of the image-BCIM. Compared with routine answer time and the number of questions (Table 5), patients had 68.3% fewer questions to answer than that of CCMQ and the time occupied by answering is reduced by 80.3%.

## 5. Discussion

Our results show that the body constitution identification model based on feature selection and classifier can simulate the doctor inquiry that pushes targeted questions to patients and the subjective patient feelings are collected by it to make further judgments on the patient's BC. The accuracy of recognizing BC automatically can be improved by combining questionnaire data with image-BC identification. Meanwhile, when collecting subjective feelings, the BCIM pushed targeted scale questions to the patients, which reduced the number of questions and the time needed to complete the questionnaire, and the identification efficiency was higher than the CCMQ. Clinically, doctors need to consider the patient's objective signs (such as imaging reports and biochemical indicators) and subjective feelings (from the doctor's consultation) to diagnose disease. According to the comparison experiment results in Tables 4 and 5, we have reason to believe that the improvement in the accuracy of BC identification is due to a comprehensive analysis of the patient's objective information and subjective feelings.

Simulating the doctor inquiry using the feature selection and the classifier can provide us with a comparatively accurate result. In practice, there are one or two key unbalanced constitutions that dominate people's current health. These key constitutions will continue to change with people's habits, the external environment, and the treatment process. When faced with a patient with a composite constitution, analyzing the key constitution that currently affects their health is key to treatment. Although the predicted result may be one of the true BC types of the patient, it provides us with a kind of opinion for reference, which helps doctors quickly pinpoint the key BC type that most affects the patient.

## 6. Conclusion

This paper adds questionnaire data to image identification and uses the CCMQ to collect subjective feelings of patients to make further judgments on BC. In order to collect the questionnaire data of the patients more fully and effectively, we propose a BC identification method based on a random generation algorithm, feature selection algorithm, and classification method. We combine the method with the image identification method to compare the accuracy of BC identification before and after the combination. The results show that our method can improve the accuracy of BC identification, effectively avoid the shortcomings of the CCMQ when collecting information and improve the efficiency of BC identification. Through the comparison experiment, we show that artificial intelligence technology in the field of medicine to achieve the level of clinical diagnosis, the collection, and comprehensive analysis of objective and subjective factors is essential. The data processed by the method in this paper include but are not limited to the problems in the CCMQ, which is of referential significance to other clinical observation scales related to physiological or pathological indicators of patients.

## Figures and Tables

**Algorithm 1:**
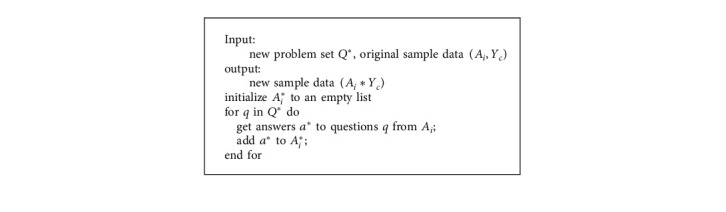
Generating new sample data.

**Table 1:** The size of the original dataset.

Constitution labels	Values
Balanced constitution	107,204
Qi-deficient constitution	94,334
Yang-deficient constitution	100,974
Yin-deficient constitution	99,785
Phlegm-dampness constitution	105,059
Damp-heat constitution	131,610
Stagnant blood constitution	114,009
Stagnant qi constitution	119,847
Inherited special constitution	127,178

**Table 2:** Experimental performances of different models.

Body constitution combination	Classifier	*k*	Acc (5-folds) (%)	Micro-averaging			Macro-averaging			Response time (s)
				Precision (%)	Recall (%)	F Score (%)	Precision (%)	Recall (%)	F Score (%)	
Balanced Constitution; Yang-deficient Constitution; Qi-deficient constitution	LDA	16	99.88	99.88	99.88	99.88	99.86	99.88	99.87	0.009
	ANN	18	99.90	99.90	99.90	99.90	99.89	99.90	99.90	0.524
	SVM	15	99.76	99.76	99.76	99.76	99.74	99.75	99.75	0.013
	RF	15	87.29	87.29	87.29	87.29	87.81	86.34	86.38	0.036
	KNN	14	97.18	97.18	97.18	97.18	97.08	97.05	97.06	134.273

Yang-deficient Constitution; Phlegm-dampness Constitution; Stagnant blood constitution	LDA	22	99.84	99.84	99.84	99.84	99.83	99.84	99.84	0.006
	ANN	25	99.81	99.81	99.81	99.81	99.81	99.82	99.81	0.389
	SVM	28	96.02	96.02	96.02	96.02	96.28	96.01	96.03	0.011
	RF	18	66.63	66.63	66.63	66.63	67.49	65.95	65.66	0.049
	KNN	22	87.48	87.48	87.48	87.48	87.45	87.50	87.46	1710.521

**Table 3:** Real discriminant results of different body constitution identification methods.

Image-BCIM	Image-BCIM + BCIM				Doctor
Result	Output-body constitution	Output-questions	Result	Time (s)	Result
Yin-deficient constitution	Yin-deficient Constitution; Qi-deficient Constitution; Balanced constitution	1. Did you get tired easily?	Qi-deficient constitution	113	Qi-deficient Constitution; Phlegm-dampness constitution
		2. Did you feel feeble when talking?			
		3. Did you suffer from shortness of breath?			
		4. Did you get palpitations?			
		5. Did you get dizziness easily or become giddy when standing up?			
		6. Did you catch colds more easily than others?			
		7. Did you prefer quietness and do not like to talk?			
		8. Did you sweat easily when you had a slightly increased physical activity?			
		9. Did the palms of your hands or soles of your feet feel hot?			
		10. Did your body and face feel hot?			
		11. Did your skin or lips feel dry?			
		12. Were your lips redder than others?			
		13. Did you get constipated easily or have dry stools?			
		14. Did you get hot flashes?			
		15. Did your eyes feel dry and use eye drops?			
		16. Did you often feel parched and need to drink water?			

**Table 4:** Comparison of methods of body constitution identification.

Method	Accuracy (%)
Image-BCIM	51.6
Image-BCIM + BCIM	77.4

**Table 5:** Comparison of methods of body constitution identification.

Method	Number of questions to be answered	Time (s)
Constitution in Chinese medicine questionnaire	60	507 ± 23
Image-BCIM	—	—
Image-BCIM + BCIM	19 ± 3	100 ± 12

## Data Availability

The data used to support the findings of this study are available from the corresponding author upon request.

## Supplementary Materials

Supplementary Materials Constitution in Chinese Medicine Questionnaire (CCMQ).

## References

[1] Li L., Yao H., Wang J., Li Y., Wang Q. (2019). The role of Chinese medicine in health maintenance and disease prevention: application of constitution theory. *The American Journal of Chinese Medicine*.

[2] Wang Q. (2005). Classification and diagnosis basis of nine basic constitutions in Chinese medicine. *Journal of Beijing University of Traditional Chinese Medicine*.

[3] Wang Qi, Yan-bo Z., Xue He-sheng (2006). Primary compiling of constitution in Chinese medicine questionnaire. *Chinese Journal of Clinical Rehabilitation*.

[4] Yan-bo Z., Wang Qi, Xue H. (2006). Preliminary assessment on performance of constitution in Chinese medicine questionnaire. *Chinese Journal of Clinical Rehabilitation*.

[5] Huang X., Zhong P., Ma G. (2017). Artificial Intelligence and Chinese medicine intelligentization. *Journal of Traditional Chinese Medicine*.

[6] Qinan Hu, Tong Yu (2018). End-to-end syndrome differentiation of yin deficiency and yang deficiency in traditional Chinese medicine. *Computer Methods and Programs in Biomedicine*.

[7] Li H., Xu B., Wang N., Liu J. Deep convolutional neural networks for classifying body constitution.

[8] Ma J. (2019). *Zero-shot Learning Method for Body Constitution Recognition Based on Tongue Image*.

[9] Er-Yang H., Gui-Hua W., Shi-Jun Z. (2017). Deep convolutional neural networks for classifying body constitution based on face image. *J. Computational and Mathematical Methods in Medicine*.

[10] Ghanbari N., Montaser Kouhsari S. (2019). A review of feature selection methods with the applications in pattern recognition in the last decade. *Fundamental Research in Electrical Engineering. Lecture Notes in Electrical Engineering*.

[11] Beatriz R., Veronica B.-C. (2019). A review of feature selection methods in medical applications. *Journal of Computers in Biology and Medicine*.

[12] Fogliatto F. S., Anzanello M. J., Soares F., Brust-Renck P. G. (2019). Decision support for breast cancer detection: classification improvement through feature selection. *Cancer Control*.

[13] Geethanjali P., Raunak V. (2018). Identification of a feature selection based pattern recognition scheme for finger movement recognition from multichannel EMG signals. *Journal of the Australasian College of Physical Scientists and Engineers in Medicine*.

[14] Dongkoo S., Im K., Jeong-Ho P. (2018). Emotional stress state detection using genetic algorithm-based feature selection on EEG signals. *International Journal of Environmental Research and Public Health*.

[15] Singh G., Singh B., Kaur M. (2019). Grasshopper optimization algorithm-based approach for the optimization of ensemble classifier and feature selection to classify epileptic EEG signals. *Medical & Biological Engineering & Computing*.

[16] Álvarez J. D., Matias-Guiu J. A., Cabrera-Martín M. N., Risco-Martín J. L., Ayala J. L. (2019). An application of machine learning with feature selection to improve diagnosis and classification of neurodegenerative disorders. *BMC Bioinformatics*.

[17] Fan H., Behdad D., Tan T. (2018). Retinal artery/vein classification using genetic-search feature selection. *Journal of Computer and Methods in Programs of Biomedecine*.

[18] Farrukh J., Ilias T., Mevludin M. (2018). A comparison of feature selection methods when using motion sensors data: a case study in Parkinson’s disease. *Journal of Conference and Proceedings in IEEE Engineering in Medicine and Biology Society*.

[19] Kang C., Huo Y., Xin L., Tian B., Yu B. (2019). Feature selection and tumor classification for microarray data using relaxed Lasso and generalized multi-class support vector machine. *Journal of Theoretical Biology*.

[20] Li F., Zhao C., Xia Z., Wang Y., Zhou X., Li G.-Z. (2012). Computer-assisted lip diagnosis on Traditional Chinese Medicine using multi-class support vector machines. *BMC Complementary and Alternative Medicine*.

[21] Wang L., Wang Y., Chang Q. (2016). Feature selection methods for big data bioinformatics: a survey from the search perspective. *Journal of Methods*.

[22] Urbanowicz R. J., Olson R. S., Schmitt P., Meeker M., Moore J. H. (2018). Benchmarking relief-based feature selection methods for bioinformatics data mining. *Journal of Biomedical Informatics*.

[23] Cömert Z., Şengür A., Budak Ü., Kocamaz A. F. (2019). Prediction of intrapartum fetal hypoxia considering feature selection algorithms and machine learning models. *Health Information Science and Systems*.

[24] Elias E., Alireza F., Mohammad S. (2019). An optimal strategy for prediction of sudden cardiac death through a pioneering feature-selection approach from HRV signal. *Journal of Computer Methods and Programs in Biomedicine*.

[25] Debasmita P., Sudarsan P., Sahoo B. (2017). Enzyme classification using multiclass support vector machine and feature subset selection. *Journal of Computers in Biological Chemistry*.

[26] Gonzalo C. G., Nicolás G.-P. (2018). Boosted feature selectors: a case study on prediction P-gp inhibitors and substrates. *Journal of Computer-Aided Molecular Design*.

[27] Yang W., Tan P., Fu X., Hong L. (2019). Prediction of amyloid aggregation rates by machine learning and feature selection. *The Journal of Chemical Physics*.

[28] Liñares B. J., Porto-Pazos Ana B., Alejandro P. (2018). Prediction of high anti-angiogenic activity peptides in silico using a generalized linear model and feature selection. *Journal of Science Reports*.

[29] Yolanda G.-C., Begonya G.-Z., Marian G.-B. (2017). Automatic migraine classification via feature selection committee and machine learning techniques over imaging and questionnaire data. *Journal of BMC Medical Informatics and Decision Making*.

[30] Eisenbarth H., Lilienfeld S. O., Yarkoni T. (2015). Using a genetic algorithm to abbreviate the psychopathic personality inventory-revised (PPI-R). *Psychological Assessment*.

[31] Sahdra B. K., Ciarrochi J., Parker P., Scrucca L. (2016). Using genetic algorithms in a large nationally representative american sample to abbreviate the multidimensional experiential avoidance questionnaire. *Frontiers in Psychology*.

[32] Enny R., Chien-Yeh H., Nurjanah N. (2019). Developing an Indonesia’s health literacy short-form survey questionnaire (HLS-EU-SQ10-IDN) using the feature selection and genetic algorithm. *Journal of Computer Methods in Programs and Biomedicine*.

[33] Hu K., Xia S., Fan M. (2017). Investigation and analysis of the TCM constitution of 53693 elderly residents in Zhongshan. *Shenzhen Journal of Integrated Traditional Chinese and Western Medicine*.

[34] Sun P., Wang J., Wang Q. (2019). Study on identification and intervention of composite constitutions. *Journal of Beijing University of Traditional Chinese Medicine*.

[35] Zhi-hui T., Qi W., Yan Z. (2019). Investigation and analysis of TCM constitution of Korean elderly group by applying Wang Qi’s nine TCM constitution questionnaire. *Guiding Journal of Traditional Chinese Medicine and Pharmacology*.

