# Supplementary material for: Personalized Body Constitution Inquiry Based on Machine Learning
Source: J Healthc Eng. 2020 Nov 12;2020:8834465. doi: 10.1155/2020/8834465 (PMC7676967; doi:10.1155/2020/8834465)
Supplement: Supplementary Materials — Constitution in Chinese Medicine Questionnaire (CCMQ). [file 8834465.f1.pdf]

Constitution in Chinese Medicine Questionnaire (CCMQ)

This questionnaire aims to survey your body constitution and to subsequently provide a reference for future health management and clinical diagnosis. Please read every question carefully and choose the most suitable item based on your actual situation or feeling in the past year. If you are unsure of the answer to a specific question, choose the answer that is most similar to your actual situation. Make sure that you answer all the questions based on your situation in the past year, (excluding the effect of drugs) and give only one answer to each question.

Explain:

【None】Never happened.

【Rarely】Occasionally happened.

【Sometimes】Sometimes it happened, but no regular pattern.

【Often】It happened most of the time.

【Always】It happened you all the time.

Experience/condition	None	Rarely	Sometimes	Often	Always
（1）Were you energetic?	1	2	3	4	5
（2）Did you get tired easily?	1	2	3	4	5
（3）Did you suffer from shortness of breath?	1	2	3	4	5
（4）Did you get palpitations?	1	2	3	4	5
（5）Did you get dizziness easily or become giddy when standing up?	1	2	3	4	5
（6）Did you prefer quietness and do not like to talk?	1	2	3	4	5
（7）Did you feel weak when talking?	1	2	3	4	5
（8）Did you forget things easily?	1	2	3	4	5
（9）Did you feel gloomy and depressed?	1	2	3	4	5
（10）Did you get anxious and worried easily?	1	2	3	4	5
（11）Did you feel sensitive, vulnerable or emotionally upset?	1	2	3	4	5
（12）Were you easily scared or frightened?	1	2	3	4	5
（13）Did you experience distention in the underarm or breast?	1	2	3	4	5
（14）Did you feel chest or stomach stuffiness?	1	2	3	4	5
（15）Did you sigh for no reason?	1	2	3	4	5
（16）Did your body feel heavy or lethargic?	1	2	3	4	5
（17）Did the palms of your hands or soles of your feet feel hot?	1	2	3	4	5
（18）Did your hands or feet feel cold or clammy?	1	2	3	4	5
（19）Did you feel cold easily in your abdomen, back, lower back or knees?	1	2	3	4	5
（20）Were you sensitive to cold and tend to wear more clothes than others?	1	2	3	4	5
（21）Did your body and face feel hot?	1	2	3	4	5
（22）Did you feel more vulnerable to the cold than others (winter coldness, air conditioners, fans, etc.)?	1	2	3	4	5
（23）Did you catch colds more easily than others?	1	2	3	4	5
（24）Did you sneeze even when you did not have a cold?	1	2	3	4	5
（25）Did you have runny or stuffy nose even when you did not have a cold?	1	2	3	4	5

Experience/condition	None	Rarely	Sometimes	Often	Always
（26）Did you cough due to seasonal change, temperature change, or unpleasant odor?	1	2	3	4	5
（27）Did you sweat easily when you had a slightly increased physical activity?	1	2	3	4	5
（28）Did you have an excessively oily forehead and/or T-zone?	1	2	3	4	5
（29）Did your skin or lips feel dry?	1	2	3	4	5
（30）Did you have allergies?(E.g. medicine, food, odors, pollen, pet dander, or during seasonal or weather change etc.)	1	2	3	4	5
（31）Did your skin get hives/urticaria easily?	1	2	3	4	5
（32）Did your skin have purpura(purple spots, ecchymosis)due to allergies?	1	2	3	4	5
（33）Did black or purple bruises appear on your skin for no reason?	1	2	3	4	5
（34）Did you skin turn red and show traces when you scratched it?	1	2	3	4	5
（35）Were your lips redder than others?	1	2	3	4	5
（36）Did you have visible capillary/thread veins on your cheeks?	1	2	3	4	5
（37）Did you feel pain somewhere in your body?	1	2	3	4	5
（38）Did you get hot flashes?	1	2	3	4	5
（39）Did your nose or your face feel greasy, oily, or shiny?	1	2	3	4	5
（40）Did you have a dark face or get brown spots easily?	1	2	3	4	5
（41）Did you get acne or sores easily?	1	2	3	4	5
（42）Did you have upper eyelid swelling?	1	2	3	4	5
（43）Did you get dark circles under the eyes easily?	1	2	3	4	5
（44）Did your eyes feel dry and use eye drops?	1	2	3	4	5
（45）Did your lips darker or purple than usual?	1	2	3	4	5
（46）Did you often feel parched and need to drink water?	1	2	3	4	5
（47）Did your throat feel strange(i.e. like something was stuck or there was a lump in your throat)?	1	2	3	4	5
（48）Did you have bitterness or a strange taste in your mouth?	1	2	3	4	5
（49）Did your mouth feel sticky?	1	2	3	4	5
（50）Did your tongue have a thick coating?	1	2	3	4	5
（51）Did you have lots of phlegm, especially in your throat?	1	2	3	4	5
（52）Did you feel uncomfortable when you drank or ate something cold, or do you avoid to drinking or eating something cold?	1	2	3	4	5
（53）Could you adapt yourself to external natural or social environment change?	1	2	3	4	5
（54）Did you suffer from insomnia?	1	2	3	4	5
（55）Did you easily contract diarrhea when you were exposed to cold or eat (or drink) something cold?	1	2	3	4	5
（56）Did you pass sticky stools and/or feel that your bowel movement is incomplete?	1	2	3	4	5
（57）Did you get constipated easily or have dry stools?	1	2	3	4	5
（58）Was your stomach/belly flabby?	1	2	3	4	5

Experience/condition	None	Rarely	Sometimes	Often	Always
（59）Did your urethral canal feel hot when you urinated, or did your urine have a dark color?	1	2	3	4	5
（60_1）Was your vaginal discharge yellowish? (only for female interviewees)	1	2	3	4	5
（60_2）Was your scrotum always wet? (only for male interviewees)	1	2	3	4	5

CCMQ scoring formula

Original scores: Sum up each item's score

Converted scores: [(original score – items)/(items*4)] *100

"R" means that the item should be scored in reverse first, namely: 1→ 5,2 → 4,3 → 3,4 → 2,5 →1

Balanced Constitution

Original scores= item1+ item2R+ item7R+ item8R+ item9R+ item22R+ item53+ item54R

Converted scores= [((item1+ item2R+ item7R+ item8R+ item9R+ item22R+ item53+ item54R)-8) /8*4] *100

Yang-deficient Constitution

Original scores= item18+ item19+ item20+ item22+ item23+ item52+ item55

Converted scores= [((item18+ item19+ item20+ item22+ item23+ item52+ item55)-7) /7*4] *100

Yin-deficient Constitution

Original scores= item17+ item21+ item29+ item35+ item38+ item44+ item46+ item57

Converted scores= [((item17+ item21+ item29+ item35+ item38+ item44+ item46+ item57)-8) /8*4] *100

Qi-deficient Constitution

Original scores= item2+ item3+ item4+ item5+ item6+ item7+ item23+ item27

Converted scores= [((item2+ item3+ item4+ item5+ item6+ item7+ item23+ item27)-8) /8*4] *100

Phlegm-dampness Constitution

Original scores= item14+ item16+ item28+ item42+ item49+ item50+ item51+ item58

Converted scores= [((item14+ item16+ item28+ item42+ item49+ item50+ item51+ item58)-8) /8*4] *100

Damp-heat Constitution

Original scores= item39+ item41+ item48+ item56+ item59+ item60_1/60_2

Converted scores= [((item39+ item41+ item48+ item56+ item59+ item item60_1/60_2)-6) /6*4] *100

Stagnant Blood Constitution

Original scores= item8+ item33+ item36+ item37+ item40+ item43+ item45

Converted scores= [((item8+ item33+ item36+ item37+ item40+ item43+ item45)-7) /7*4] *100

Stagnant Qi Constitution

Original scores= item9+ item10+ item11+ item12+ item13+ item15+ item47

Converted scores= [((item9+ item10+ item11+ item12+ item13+ item15+ item47)-7) /7*4] *100

Inherited Special Constitution

Original scores= item24+ item25+ item26+ item30+ item31+ item32+ item34

Converted scores= [((item24+ item25+ item26+ item30+ item31+ item32+ item34)-8) /8*4] *100

Determination:

Body Constitution	Condition	Result
Balanced Constitution	Converted scores $\geqslant$ 60	Yes
	The rest constitutions' converted scores all $<$ 30	
	Converted scores $\geqslant$ 60	Basically yes
	The rest constitutions' converted scores all $<$ 40	
	Not stratified the condition above	No
Unbalanced Constitution	Converted scores $\geqslant$ 40	Yes
	Converted scores 30~39	Tend to
	Converted scores $<$ 30	No
